# NETO2 promotes invasion and metastasis of gastric cancer cells via activation of PI3K/Akt/NF-κB/Snail axis and predicts outcome of the patients

**DOI:** 10.1038/s41419-019-1388-5

**Published:** 2019-02-15

**Authors:** Jun-yan Liu, Lei Jiang, Tao He, Jia-jia Liu, Jun-yan Fan, Xian-hui Xu, Bo Tang, Yan Shi, Yong-liang Zhao, Feng Qian, Yan Wang, You-hong Cui, Pei-wu Yu

**Affiliations:** 1 Department of General Surgery and Center of Minimal Invasive Gastrointestinal Surgery, Southwest Hospital, Third Military Medical University (Army Medical University), 400038 Chongqing, China; 2 Institute of Pathology and Southwest Cancer Center, and Key Laboratory of Tumor Immunopathology of Ministry of Education of China, Southwest Hospital, Third Military Medical University (Army Medical University), 400038 Chongqing, China

## Abstract

Aberrant expression of neuropilin and tolloid-like 2 (NETO2) has been observed during the progression of some human carcinomas. However, the expression pattern and clinical relevance of NETO2 in gastric cancer (GC) remain to be elucidated. In this study, we found that NETO2 expression was higher in GC tissues compared with paired non-cancerous tissues. Moreover, the expression of NETO2 was positively correlated with clinical stage, invasion depth, lymph node metastasis, and tumor size, but inversely correlated with overall and disease-free survival rates. Cox regression analysis identified NETO2 as an independent prognostic indicator for GC patients. Overexpression of NETO2 facilitated migration and invasion of GC cells in vitro and metastasis in vivo in association with induction of epithelial-mesenchymal transition. Conversely, knockdown of NETO2 had the opposite effects. Mechanistically, silencing NETO2 reduced the phosphorylation of PI3K, AKT, and NF-κB p65 as well as the expression of Snail, whereas NETO2 overexpression achieved the opposite results. Furthermore, we identified TNFRSF12A as a mediator for NETO2 to activate PI3K/AKT/NF-κB/Snail axis. Collectively, our results demonstrate that NETO2 promotes invasion and metastasis of GC cells and represents a novel prognostic indicator as well as a potential therapeutic target in GC.

## Introduction

Gastric cancer (GC) is the fifth most common cancer and the third leading cause of cancer-related deaths worldwide^1,2^. Currently, the progress of comprehensive therapeutic strategies has greatly improved the treatment effect of GC patients. However, the prognosis of most GC patients is still poor, mainly due to advanced stage of disease at diagnosis and limited understanding of the molecular mechanisms underlying the invasion and metastasis of GC^3,4^. Therefore, a better insight into the molecular basis for invasion and metastasis of GC would facilitate the development of more effective therapeutic strategies for the patients.

Neuropilin and tolloid-like 2 (NETO2), a member of the subfamily of CUB domain and LDLa-containing proteins^5^, was identified as an auxiliary protein of neuronal kainate receptors (KARs)^6,7^, and played critical roles in regulating the functions of KARs^8,9^. It was also able to bind to the active oligomeric form of K^+^-Cl^−^ cotransporter (KCC2) to enhance its recycling in hippocampal neurons^10,11^. Recently, elevated mRNA levels of NETO2 were detected in several types of tumors^12,13^. In patients with colorectal cancer (CRC), NETO2 upregulation was significantly correlated with advanced TNM stages and poor survival^14^. In hepatocellular carcinoma, NETO2 has been identified as a member of the five-gene transcriptomic signature which predicted poor outcome of the patients^15^. However, little is known about the expression pattern and role of NETO2 in GC.

In the current study, we found that NETO2 was significantly upregulated in GC tissues and its expression level was closely associated with the clinicopathological parameters and overall and disease-free survival rates of the patients. NETO2 enhanced the invasive ability of GC cells in vitro and metastatic capability in vivo by inducing epithelial–mesenchymal transition (EMT) through upregulating TNFRSF12A to activate PI3K/AKT/NF-κB/Snail axis. Thus, NETO2 is a tumor-promoting factor in GC and may serve as a novel prognostic indicator as well as a potential therapeutic target for GC.

## Results

### NETO2 is upregulated in GC tissues and associated with clinicopathological features of the patients

**Fig. 1:**
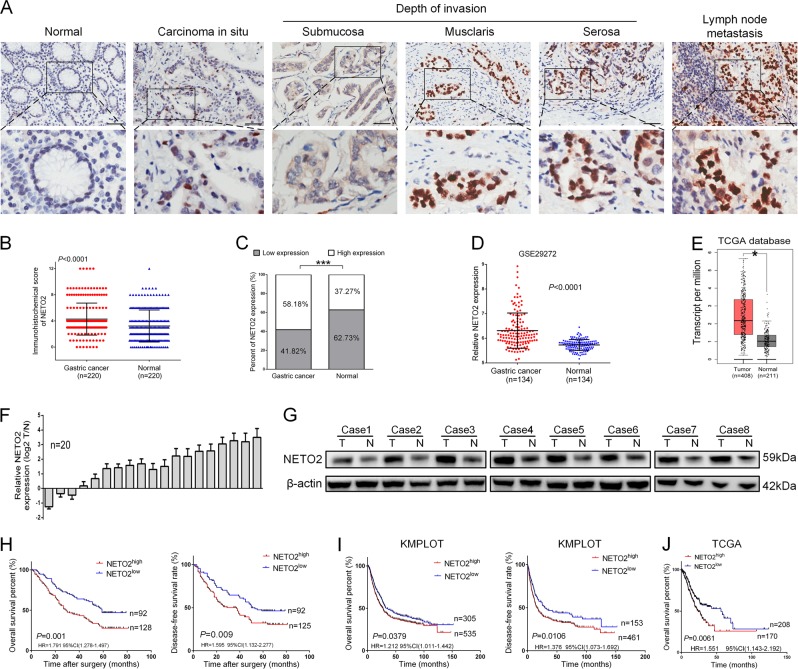
NETO2 is highly expressed in gastric cancer tissues and correlated with outcome of the patients. **a** Representative immunohistochemical staining (IHC) images of NETO2 in adjacent normal tissue, gastric cancer tissues with different invasion depth and metastatic lymph node. Scale bar = 50 μm. **b** The IHC score of NETO2 in gastric cancer tissues was significantly higher than that in adjacent normal tissues. **c** High expression of NETO2 was more frequent in gastric cancer tissues than in adjacent normal tissues. **d** Data in GEO GSE29272 showed higher mRNA level of NETO2 in gastric cancer tissues compared to adjacent normal tissues. **e** Data in TCGA database showed higher mRNA level of NETO2 in gastric cancer tissues compared to adjacent normal tissues. **f** mRNA levels of NETO2 were higher in 20 fresh surgical gastric tumor specimens (t) than that in paired adjacent normal tissues (n). **g** NETO2 protein levels were higher in eight fresh surgical gastric cancer (t) tissues than that in adjacent normal (n) tissues. **h** Kaplan–Meier estimation indicated significantly lower overall and disease-free survival rates in patients with NETO2^high^ gastric cancer than that in NETO2^low^ patients. **i** Data in KMPLOT database showed that NETO2^high^ patients had significantly lower overall and disease-free survival rates than that in NETO2^low^ patients. **j** Data in TCGA database showed that NETO2^high^ patients had significantly lower overall survival rate than that in NETO2^low^ patients. ^*^p < 0.05, ^***^p < 0.001

NETO2 expression was examined in 220 GC samples and paired adjacent non-tumor tissues by immunohistochemistry (IHC). The staining of NETO2 was significantly higher in cancer tissues and metastatic lymph nodes than that in normal gastric mucosa (*p* *<* 0.0001) (Fig. 1a, b). High expression of NETO2 (NETO2^high^) was more frequent in GC tissues (128/220, 58.18%) compared with adjacent non-tumor tissues (82/220, 37.27%) (*p* *<* 0.001) (Fig. 1c). These results were supported by analysis on six individual datasets in NCBI GEO database, including GSE29272 (*p* *<* 0.0001, Fig. 1d), GSE65801 (*p* *=* 0.0002, Fig. S1A), GSE63089 (*p* < 0.0001, Fig. S1B), GSE54129 (*p* = 0.0002, Fig. S1C), GSE27342 (*p* < 0.0001, Fig. S1D), and GSE13911 (*p* < 0.0001, Fig. S1E) as well as on TCGA database (*p* < 0.05, Fig. 1e). We also detected NETO2 mRNA in 20 pairs of fresh GC tissues by qRT-PCR as well as NETO2 protein in 8 pairs of fresh GC tissues through western blotting. Both mRNA and protein levels of NETO2 were significantly elevated in tumor tissues compared to adjacent non-tumor counterparts (Fig. 1f, g). These results indicate that NETO2 is highly expressed in GC tissues.

Next, we analyzed the correlation between NETO2 expression and clinicopathological features of patients with GC. The protein level of NETO2 was markedly correlated with TNM stage (*p* = 0.004), tumor invasion depth (*p* = 0.016), lymph node metastasis (*p* = 0.022), and tumor size (*p* *=* 0.045) (Table 1). Kaplan–Meier survival analysis further showed that NETO2^high^ patients had a significantly lower overall survival (OS, *p* = 0.001) and disease-free survival (DFS, *p* = 0.009) rates than that of NETO2^low^ patients (Fig. 1h). Univariate and multivariate Cox regression analyses revealed that NETO2 expression was an independent prognostic factor for OS (*p* = 0.001 and 0.011, respectively) and DFS (*p* = 0.006 and 0.041, respectively) in GC patients (Tables 2 and  3). Analyses on GC data from KMPLOT and TCGA databases also supported that high NETO2 expression acted as an indicator for poor survival of GC patients (Fig. 1i, j). Thus, these results reveal that NETO2 may play oncogenic roles in GC.

**Table 1:** The relationship between NETO2 expression and clinicopathological features of patients with gastric cancer

Clinicopathological features		NETO2 expression			
	Number	Low	High	*χ* ^2^	*P* value
Age (years)				0.748	0.387
≥60	79	30	49		
<60	141	62	79		
Sex				0.298	0.585
Male	151	65	86		
Female	69	27	42		
Histological grade				0.641	0.424
G1 + G2	70	32	38		
G3	150	60	90		
TNM stage				8.368	**0.004**
I/II	111	57	54		
III/IV	109	35	74		
T stage				5.773	**0.016**
T1–T2	58	32	26		
T3–T4	162	60	102		
N stage				5.284	**0.022**
N0	72	38	34		
N1–N3	148	54	94		
M stage				2.533	0.112
M0	203	88	115		
M1	17	4	13		
Tumor size (cm)				4.037	**0.045**
<5	146	68	78		
≥5	74	24	50		
Tumor location				0.529	0.467
Distal gastric	118	52	66		
Proximal gastric	102	40	62

**Table 2:** Univariate and multivariate analyses of overall survival in patients with gastric cancer

Prognostic variables	Univariate analysis		Multivariate analysis	
	HR (95% CI)	*P* value	HR (95% CI)	*P* value
Age (years)	1.096 (0.774–1.552)	0.606	–	–
Sex	0.745 (0.515–1.078)	0.119	–	–
Histological grade	0.779 (0.549–1.105)	0.161	–	–
TNM stage	2.360 (1.673–3.330)	**0.000**	1.040 (0.619–1.748)	0.882
T stage	2.505 (1.620–3.874)	**0.000**	1.636 (0.972–2.755)	0.064
N stage	2.238 (1.516–3.305)	**0.000**	1.635 (0.994–2.691)	0.053
M stage	4.292 (2.514–7.328)	**0.000**	3.199 (1.822–5.619)	**0.000**
Tumor location	0.688 (0.492–0.963)	**0.029**	0.940 (0.660–1.339)	0.733
Tumor size	1.989 (1.409–2.807)	**0.000**	1.356 (0.934–1.970)	0.109
NETO2 expression	1.800 (1.265–2.560)	**0.001**	1.588 (1.109–2.273)	**0.011**

**Table 3:** Univariate and multivariate analyses of disease-free survival in patients with gastric cancer

Prognostic variables	Univariate analysis		Multivariate analysis	
	HR (95% CI)	*P* value	HR (95% CI)	*P* value
Age (years)	1.005 (0.700–1.444)	0.977	–	–
Sex	0.782 (0.536–1.139)	0.200	–	–
Histological grade	0.866 (0.601–1.249)	0.442	–	–
TNM stage	2.187 (1.537–3.111)	**0.000**	0.996 (0.583–1.703)	0.989
T stage	2.338 (1.507–3.627)	**0.000**	1.589 (0.939–2.687)	0.084
N stage	2.086 (1.407–3.093)	**0.000**	1.587 (0.955–2.639)	0.075
M stage	3.820 (2.163–6.747)	**0.000**	2.857 (1.572–5.129)	**0.001**
Tumor location	0.651 (0.461–0.920)	**0.015**	0.850 (0.590–1.223)	0.381
Tumor size	1.882 (1.317–2.690)	**0.001**	1.302 (0.885–1.915)	0.181
NETO2 expression	1.652 (1.154–2.364)	**0.006**	1.463 (1.016–2.107)	**0.041**

### NETO2 promotes migration, invasion, and metastasis of GC cells in association with induction of EMT

**Fig. 2:**
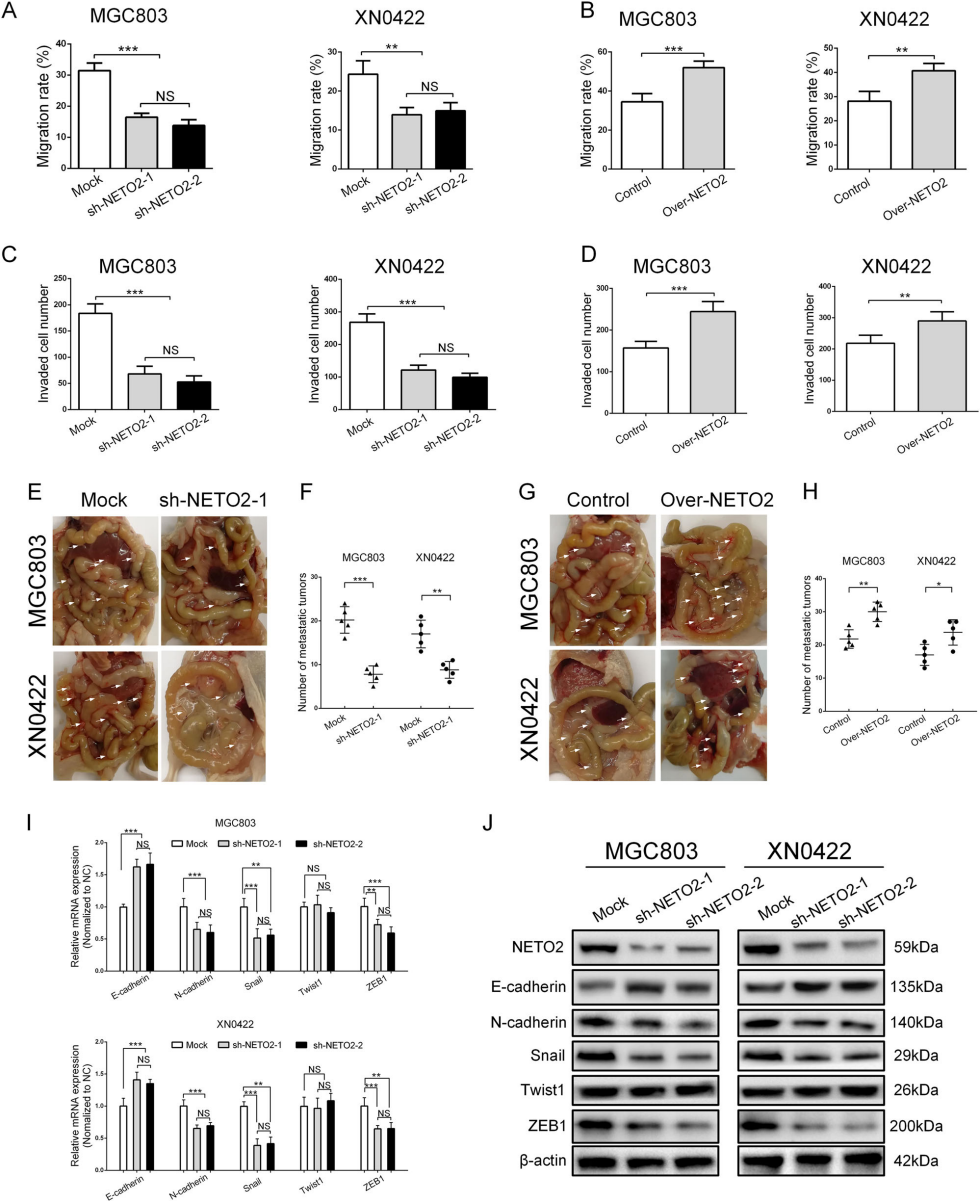
NETO2 promotes the migration and invasion of gastric cancer cells in vitro and metastasis in vivo in association with epithelial–mesenchymal transition (EMT). **a** Quantification of wound healing assay showed decreased migration ability in NETO2-knockdown cells compared to mock cells. **b** Quantification of wound healing assay showed increased migration ability in Over-NETO2 cells compared to control cells. **c** Quantification of transwell invasion assay showed decreased invasive ability in NETO2-knockdown cells compared to mock cells. **d** Quantification of transwell invasion assay showed increased invasive ability in Over-NETO2 cells compared to control cells. **e** Representative images of intraperitoneal metastasis assay showed metastatic nodules derived from sh-NETO2–1 cells and mock cells. Arrows indicate intraperitoreal nodules. **f** Quantification of intraperitoneal metastasis assay showed reduced number of metastatic foci formed by NETO2-knockdown cells compared to mock cells. **g** Representative images of intraperitoneal metastasis assay showed metastatic nodules derived from Over-NETO2 and control cells. Arrows indicate intraperitoreal nodules. **h** Quantification of intraperitoneal metastasis assay showed increased number of metastatic foci formed by Over-NETO2 cells compared to control cells. **i** qRT-PCR analysis showed significantly upregulated E-cadherin and downregulated N-cadherin, Snail and ZEB1 at mRNA level in NETO2-knockdown cells compared to mock cells. **j** Western blotting analysis showed significantly upregulated E-cadherin and downregulated Fibronectin, N-cadherin, Snail, and ZEB1 at protein level in NETO2-knockdown cells compared to mock cells. ^*^p < 0.05, ^**^p < 0.01, ^***^p < 0.001, NS no significant

To further evaluate the function of NETO2, we first examined the expression of NETO2 in a gastric epithelial cell line (GES-1) and 5 different GC cell lines (SGC7901, BGC823, MKN-45, AGS, and MGC803) as well as a primary GC cell line (XN0422) by qRT-PCR and western blotting, respectively (Fig. S2A and S2B). MGC803 and XN0422 showed relatively higher levels of NETO2 expression and were used to establish the cell models of NETO2-knockdown and -overexpression (Fig. S3A, S3B and S3C). Since our clinical findings pointed the involvement of NETO2 in the invasion and metastasis of GC, we addressed this issue with the NETO2 gene-manipulated cell models. Compared with mock cells, silencing NETO2 significantly decreased the migratory capabilities (Fig. 2a and Fig. S4A), while overexpression of NETO2 markedly enhanced the migratory abilities (Fig. 2B and Fig. S4B). Similarly, the invasion of GC cells was attenuated by depletion of NETO2 but enhanced by forced expression of NETO2 (Fig. 2c, d and Fig. S4C and S4D). In the intraperitoneal metastasis model, the metastatic nodules were significantly fewer in mice implanted with NETO2-knockdown cells than that implanted with mock cells (*p* < 0.001 for MGC803 and *p* < 0.01 for XN0422) (Fig. 2e, f), whereas mice implanted with Over-NETO2 cells formed more metastatic nodules compared to the mice implanted with control cells (*p* < 0.01 for MGC803 and *p* < 0.05 for XN0422) (Fig. 2g, h). HE staining further confirmed that the metastatic nodules were derived from GC cells (Fig. S4E). Together, these results suggest that NETO2 enhances the migration and invasion and metastasis of GC cells.

It has been well known that invasion and metastasis of GC cells largely rely on EMT^16^, which promoted us to investigate whether EMT was involved in NETO2-induced invasion and metastasis of GC cells. As expected, knockdown of NETO2 led to inhibition of EMT, featured with upregulation of E-cadherin, downregulation N-cadherin, Snail, and ZEB1 (Fig. 2i, j). We also observed that NETO2 knockdown altered the GC cells from mesenchymal-like morphology to epithelial morphology (Fig. S5A), whereas overexpression of NETO2 turned GC cells into more spindle-shaped morphology with more protrusions (Fig. S5B). Therefore, these results suggest that EMT might mediate NETO2-enhanced invasion and metastasis in GC cells.

### NETO2 activates PI3K/AKT pathway to induce EMT in GC cells

**Fig. 3:**
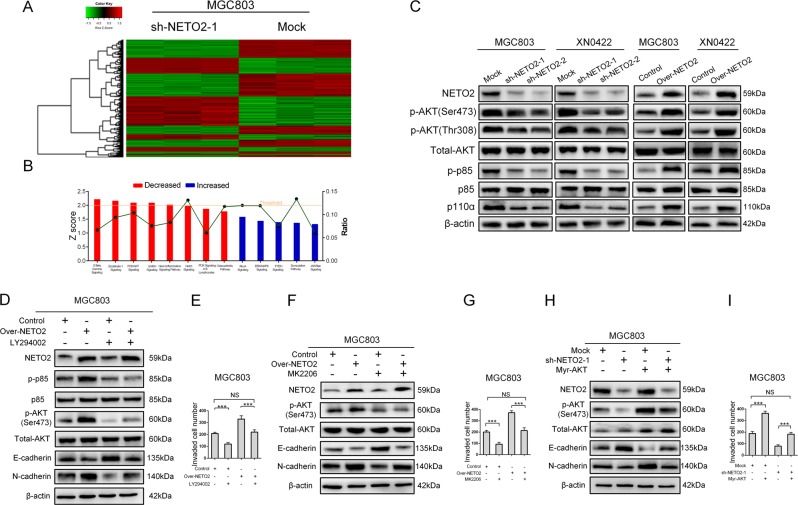
NETO2 activates PI3K/AKT pathway to induce EMT in GC cells. **a** Microarray heatmap showed differentially expressed genes in mock and sh-NETO2–1 cells. The color key for the normalized expression data was shown at the top of the microarray heatmap (green means downregulated genes and red represents upregulated genes). **b** Ingenuity Pathway Analysis (IPA) of the most significantly changed classical pathways after knockdown of NETO2 in gastric cancer cells. All signaling pathways were sorted according to the *z* score (left *y*-axis). The ratio represented the relative number of differentially expressed genes in a specified signaling pathway (right *y*-axis). **c** Western blotting analysis showed reduced phosphorylation levels of AKT and p85 in sh-NETO2–1 cells, while increased phosphorylation levels of AKT and p85 in Over-NETO2 cells compared to their control cells, respectively. **d** Western blotting analysis showed that treatment with LY294002 (10 μM) attenuated NETO2-enhanced phosphorylation of AKT and inhibited NETO2-induced EMT in Over-NETO2 cells. **e** Quantification of transwell invasion assay showed that treatment with LY294002 (10 μM) attenuated the enhanced invasive ability in Over-NETO2 cells. **f** Western blotting analysis showed that treatment with MK2206 (5 μM) attenuated NETO2-enhanced phosphorylation of AKT and inhibited NETO2-induced EMT in Over-NETO2 cells. **g** Quantification of transwell invasion assay showed that treatment with MK2206 (5 μM) attenuated the invasive ability in Over-NETO2 cells. **h** Western blotting analysis showed that transfection with myristoylated (myr) -AKT increased the phosphorylation level of AKT and induced EMT in NETO2-knockdown cells. **i** Quantification of transwell invasion assay showed that transfection with myr-AKT increased the invasive ability in NETO2-knockdown cells. ^***^p<0.001; NS no significant

Then, we wanted to study the signaling pathways responsible to regulate NETO2-promoted invasion and metastasis of GC cells. For this purpose, we performed gene expression profiling using sh-NETO2–1 and its control (mock) cells. A total of 1243 genes (|log FC| ≥ 1.5 and FDR < 0.05) were identified as differentially expressed genes, including 580 upregulated genes and 663 downregulated genes (Fig. 3a). Ingenuity Pathway Analysis (IPA) revealed that knockdown of NETO2 affected a wide range of cellular functions and human diseases. The most significantly enriched disease was cancer and the most affected cellular functions were invasion, migration, and EMT of tumor cells (Fig. S6A and S6B). IPA also indicated that PI3K/AKT pathway was one of the most enriched pathways after knockdown of NETO2 (*z* score = −2.111, *p* = 0.0269) (Fig. 3b). Thus, we examined the phosphorylation levels of both PI3K (p85) and AKT in NETO2-knockdown and overexpressed GC cells. Knockdown of NETO2 significantly inhibited the phosphorylation of p85 and AKT compared to mock cells, while overexpression of NETO2 markedly increased the phosphorylation of p85 and AKT (Fig. 3c). Treatment with LY294002, a specific inhibitor for PI3K, significantly abrogated the enhanced invasive ability and phosphorylation of AKT, as well as upregulated N-cadherin and downregulated E-cadherin induced by overexpression of NETO2 (Figs 3d, e; Fig. S7A). Similar results were observed when treated with MK2206, an AKT inhibitor (Fig. 3f, g; Fig. S7B). Exogenous expression of myristoylated AKT (myr-AKT), a constitutive activation form of AKT, reversed the inhibited invasive ability, downregulated N-cadherin, and phosphorylation of AKT, as well as, upregulated E-cadherin induced by knockdown of NETO2 (Fig. 3h, i; Fig. S7C). These results suggest that PI3K/AKT signaling pathway participates in NETO2-induced EMT in GC cells.

### PI3K/AKT-activated NF-κB/Snail axis contributes to the NETO2 function in GC

**Fig. 4:**
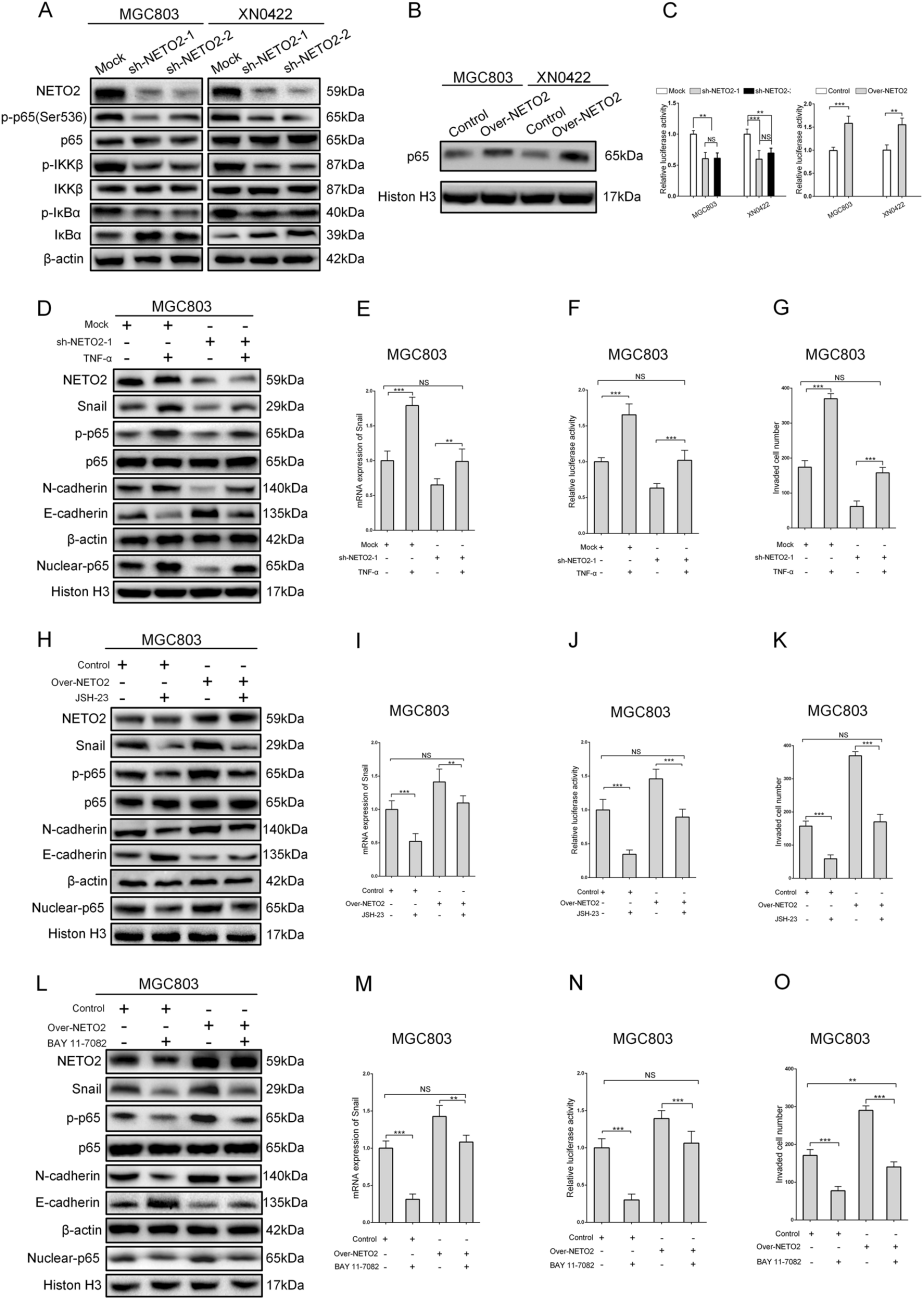
PI3K/AKT activates NF-κB/Snail axis to regulate NETO2-induced EMT. **a** Western blotting analysis showed attenuated phosphorylation of p65, IKKβ, and IκBα in NETO2-knockdown cells compared to mock cells. **b** Western blotting analysis showed increased nuclear accumulation of NF-κB p65 in Over-NETO2 cells compared to control cells. **c** The NF-κB transcription activity was decreased in NETO2-knockdown gastric cancer cells, while increased in NETO2-overexpression gastric cancer cells. **d** Western blotting analysis showed that TNF-α (10 ng/mL) treatment resulted in increased phosphorylation and nuclear accumulation of p65, downregulated E-cadherin and upregulated N-cadherin in sh-NETO2–1 cells. **e** The mRNA level of Snail was upregulated after treatment with TNF-α (10 ng/mL) in sh-NETO2–1 GC cells. **f** Treatment with TNF-α (10 ng/mL) enhanced NF-κB transcription activity measured by dual luciferase assay in sh-NETO2–1 GC cells. **g** Quantification of transwell invasion assay showed increased invasive ability after treatment with TNF-α (10 ng/mL) in sh-NETO2–1 GC cells. **h** Western blotting analysis showed that JSH-23 (10 μM) treatment led to decreased phosphorylation and nuclear accumulation of p65, upregulated E-cadherin, and downregulated N-cadherin in Over-NETO2 cells. **i** The mRNA level of Snail was decreased after treatment with JSH-23 (10 μM) in Over-NETO2 GC cells. **j** Treatment with JSH-23 (10 μM) decreased the NF-κB transcription activity measured by dual luciferase assay in Over-NETO2 GC cells. **k** Quantification of transwell invasion assay showed decreased invasive ability after treatment with JSH-23 (10 μM) in Over-NETO2 GC cells. **l** Western blotting analysis showed that BAY 11–7082 (10 μM) treatment resulted in decreased phosphorylation and nuclear accumulation of p65, upregulated E-cadherin and downregulated N-cadherin in Over-NETO2 cells. **m** The mRNA level of Snail was decreased after treatment with BAY 11–7082 (10 μM) in Over-NETO2 GC cells. **n** Treatment with BAY 11–7082 (10 μM) decreased the NF-κB transcription activity measured by dual luciferase assay in Over-NETO2 GC cells. **o** Quantification of transwell invasion assay showed decreased invasive ability after treatment with BAY 11–7082 (10 μM) in Over-NETO2 GC cells. ^**^p < 0.01, ^***^p < 0.001; NS no significant

It is well known that PI3K/AKT activates NF-κB signaling pathway in many cancers^17,18^. As shown in Fig. 4a, knockdown of NETO2 suppressed the phosphorylation of NF-κB p65, IKKβ, and IκBα. Furthermore, overexpression of NETO2 significantly increased the nuclear accumulation of p65 in MGC803 and XN0422 cells (Fig. 4b). Luciferase assay showed that overexpression of NETO2 significantly enhanced NF-κB transcription activity, while knockdown of NETO2 led to an opposite result (Fig. 4c). Additionally, NF-κB is known to function as a direct transcriptional factor for Snail^19–21^, and therefore, we explored whether PI3K/AKT/NF-κB/Snail axis was implicated in NETO2-induced EMT in GC cells. Treatment with tumor necrosis factor-α (TNF-α), a typical cytokine to activate NF-κB^19^, significantly rescued the inhibitory effects of NETO2 knockdown on Snail expression and p65 phosphorylation (Fig. 4d, e). TNF-α treatment also restored the transcription activity of NF-κB and invasive capability in NETO2-knockdown cells (Fig. 4f, g; Fig. S7D). Treatment with JSH-23, an inhibitor of NF-κB, suppressed NETO2-induced EMT, Snail expression, transcription activity of NF-κB, and invasive capability in NETO2 overexpression cells (Fig. 4h–k; Fig. S7E). Treatment with Bay 11–7082, an IKK inhibitor, showed similar results with JSH-23 treatment (Fig. 4l–o; Fig. S7F). Thus, PI3K/AKT/NF-κB/Snail axis is closely involved in the function of NETO2 in GC cells.

### TNF receptor superfamily member 12A (TNFRSF12A) mediates NETO2-induced activation of PI3K/AKT/NF-κB/Snail axis in GC cells

**Fig. 5:**
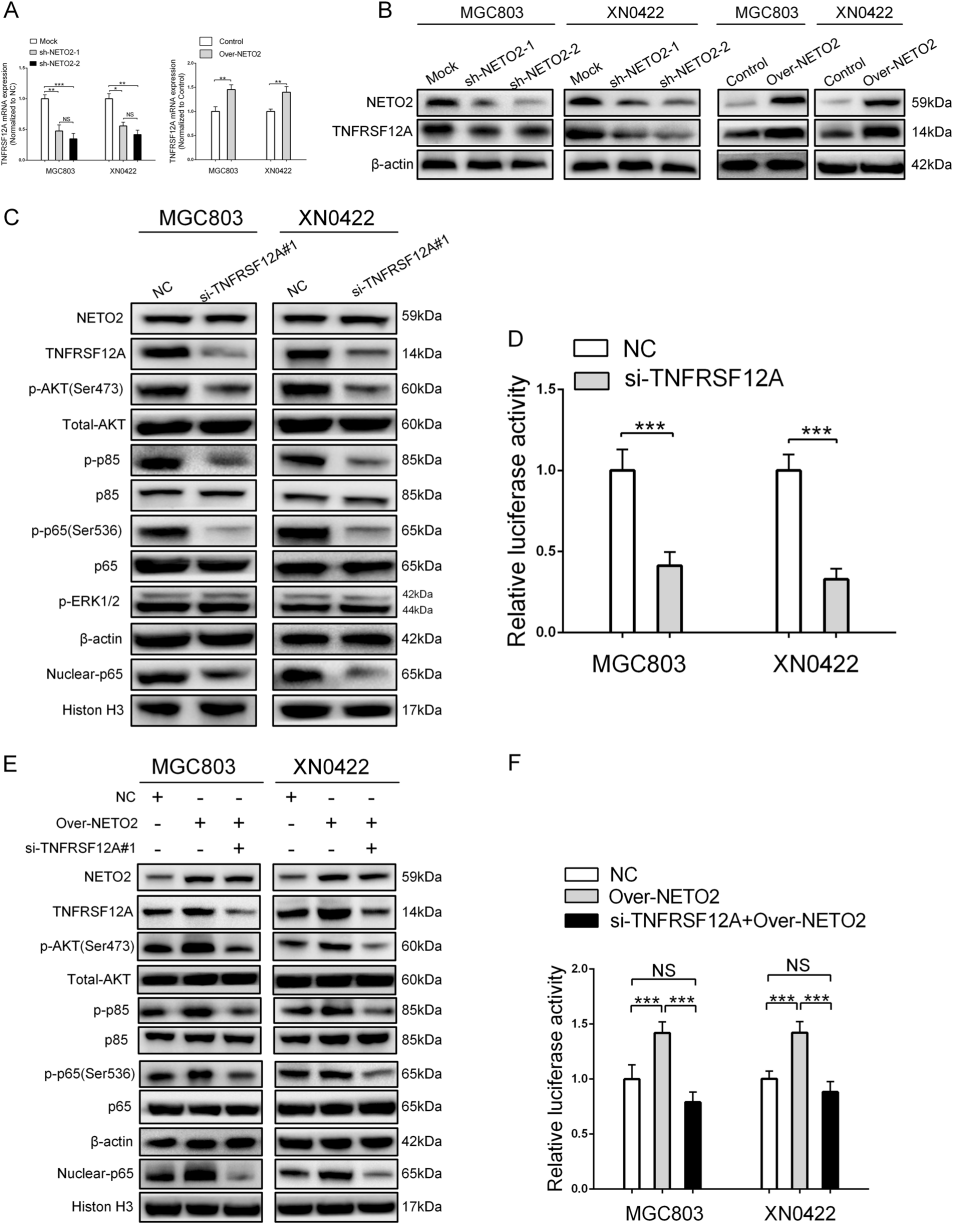
TNFRSF12A mediates NETO2-induced activation of PI3K/AKT/NF-κB/Snail axis. **a** qRT-PCR analysis showed knockdown of NETO2 downregulated the mRNA level of TNFRSF12A, while overexpression of NETO2 upregulated the mRNA level of TNFRSF12A. **b** Western blotting analysis showed that depletion of NETO2 downregulated TNFRSF12A expression, while overexpression of NETO2 upregulated TNFRSF12A expression. **c** Depletion of TNFRSF12A in MGC803 and XN0422 cells inactivated PI3K/AKT/NF-κB pathway, but had no influence on Erk1/2 pathway. **d** Depletion of TNFRSF12A impaired the NF-κB transcription activity in MGC803 and XN0422 cells. **e** Silencing TNFRSF12A abrogated NETO2-induced activation of PI3K/AKT/NF-κB pathway. **f** Depletion of TNFRSF12A inhibited NETO2-induced NF-κB transcription activity; ^*^p < 0.05, ^**^p < 0.01, ^***^p < 0.001; NS no significant

Dysregulation of TNFRSF12A has been demonstrated to play a crucial role in many human malignancies^22^ and was reported to regulate PI3K/AKT^23,24^ and NF-κB^25^ pathways. Interestingly, our results of IPA showed that TNFRSF12A was significantly downregulated after knockdown of NETO2 (fold change = −3.03, *p* < 0.0001, Supplementary File 1). Accordingly, we detected expression of TNFRSF12A at both mRNA and protein levels after manipulation of NETO2 gene. TNFRSF12A was markedly downregulated by NETO2 knockdown and significantly upregulated by NETO2 overexpression (Fig. 5a, b). Depletion of TNFRSF12A in MGC803 and XN0422 cells (Fig. S8A and S8B) impaired the phosphorylation of AKT, p85, and p65 as well as nuclear accumulation of p65 (Fig. 5c). Furthermore, the NF-κB transcription activity in siTNFRSF12A cells was significantly inhibited (Fig. 5d). Depletion of TNFRSF12A blocked NETO2-induced phosphorylation of AKT, p85, and p65 as well as the nuclear accumulation of p65 (Fig. 5e). Luciferase assay showed the requirement of TNFRSF12A for NETO2-activating NF-κB signaling (Fig. 5f). These results indicate that TNFRSF12A is also affected by NETO2 and might mediate the activation of PI3K/AKT/NF-κB/Snail axis by NETO2.

## Discussion

The role and clinical relevance of NETO2 in cancers have not been well illustrated. However, a few studies have suggested that NETO2 is a tumor-promoting molecule. In prostate cancer, the expression level of NETO2 was significantly correlated with intratumor heterogeneity in association with PTEN deletion^26^. Christine et al.^27^ found that NETO2 was significantly downregulated after exogenous overexpression of Nm23-H1, a metastatic suppressor gene, in breast carcinoma cell lines. In the present study, we demonstrated that NETO2 was an important molecule enhancing the invasion and metastasis of GC, and elevated NETO2 was an indicator for disease progression and poor survival of patients with GC. To the best of our knowledge, this is the first study to reveal the roles and clinical relevance of NETO2 in GC.

EMT is a central step in the invasion and metastasis of various cancers^28^, and 90% of tumors show different degrees of EMT during tumor development including GC^29–31^. During EMT, epithelial cells lose polarities and convert to a mesenchymal morphology accompanied by reduction of epithelial markers and upregulation of mesenchymal markers, resulting in increased migratory and invasive abilities^32,33^. In our work, manipulation of NETO2 gene significantly changed not only the cell morphology but also the expression of mesenchymal and epithelial markers in GC cells, implying that induction of EMT was involved in NETO2-promoted invasion and metastasis of GC cells. However, Maria et al.^34^ reported that the elevated mRNA level of NETO2 in CRC was not directly correlated with expression of EMT-related genes. The reason for the discrepant results is not clear but may be may be attributed to the difference in tumor types. Another reason might be post-transcriptional and post-translational regulation. Therefore, our data provide a novel evidence on NETO2 function in GC cells.

It is well known that PI3K/AKT pathway plays an important role in promoting invasion and metastasis by regulating EMT in numerous cancers^35^. In addition, NF-κB is an important downstream target of PI3K/AKT signaling capable of inducing EMT^36–39^. Consistently, we demonstrated that PI3K/AKT pathway was critical in NETO2-induced EMT, and also responsible for NETO2-induced activation NF-κB in GC cells.

EMT involves transcriptional reprogramming mediated by the activation of various transcription factors, including such as TWIST1/2, Snail, Slug, and ZEB1/2, which suppress the expression of key epithelial markers and upregulate mesenchymal markers either directly or indirectly^40^. In this study, we found that Snail was the most important EMT-related transcription factor involved in NETO2-induced EMT. Snail belongs to the family of zinc-finger transcription factors, which functions to initiate the EMT process through binding to E-box motifs in the promoter region of E-cadherin^41^. It has been proved that NF-κB acts as a regulator of Snail in the process of EMT via transcriptional and post-translational mechanisms^42,43^. Our work demonstrated that upregulation of Snail by NF-κB contributed to NETO2-induced EMT in GC cells.

Finally, our work revealed that NETO2-regulated PI3K/AKT pathway was mediated by TNFRSF12A. TNFRSF12A, also known as fibroblast growth factor-inducible 14 (Fn14), is a type I transmembrane protein functioning as a receptor for TNFRSF12 (TWEAK)^44^. It has been reported that TNFRSF12A is dramatically overexpressed in many tumors^45–47^ and promotes multiple cellular processes including proliferation, invasion, apoptosis, and angiogenesis^22,48–50^. It has also been demonstrated that TNFRSF12A is able to activate PI3K/AKT signaling by phosphorylating PI3K^51^ and/or AKT^45^. Moreover, TNFRSF12A activates canonical and non-canonical NF-κB pathways via TRAF2^52,53^. Thus, our data revealed that TNFRSF12A/PI3K/Akt/NF-κB/Snail axis was the main mechanism of NETO2-induced invasion and metastasis of GC cells.

In conclusion, the current study demonstrated that high levels of NETO2 expression in GC tissues correlated with poor prognosis of the patients. NETO2 was an important onco-protein that might activate TNFRSF12A/PI3K/AKT/NF-κB/Snail axis to induce EMT and consequently promote the invasion and metastasis in GC cells. Thus, NETO2 might serve as a new prognostic indicator and a potential therapeutic target for GC.

## Materials and methods

### Patients and tissue specimens

Tumor and adjacent normal tissues were obtained from 220 GC patients who underwent radical gastrectomy in Southwest Hospital (Chongqing, China) between 2007 and 2010. None of the patients were treated with immunotherapy, radiotherapy, or chemotherapy before surgery. The clinicopathological information of all the subjects was available in Table S1. TNM stage was classified according to the 8th edition of American Joint Committee on Cancer (AJCC) staging system^54^. All data were analyzed anonymously and all experiments were performed in compliance with the Helsinki Declaration. The study was approved by the Ethics Committee of Southwest Hospital and the informed consent was obtained from all patients.

### Cell culture and treatments

The human GC cell lines MGC803, BGC823, MKN-45, SGC7901, AGS, and the immortalized gastric epithelium cell line (GES-1) were purchased from the Shanghai Institute of Biochemistry and Cell Biology, Chinese Academy of Sciences (Shanghai, China). Primary GC cell line XN0422 was established in our laboratory^55^. Cell culture was conducted as previously described^56^. The cells were treated as follows: PI3K inhibitor LY294002 (CST) 10 μM for 24 h; AKT inhibitor MK2206 (Selleck Chemicals) 5 μM for 24 h; NF-κB inhibitor JSH-23 (Abcam) 10 μM for 24 h; IKK inhibitor BAY 11–7082 (Abcam) 10 μM for 12 h; NF-κB activator TNF-α (Sigma-Aldrich, USA) 10 ng/mL for 24 h.

### Immunohistochemistry

IHC staining was conducted as previously described^57^. A rabbit polyclonal antibody against NETO2 (ab171651, 1:100, Abcam, Cambridge, UK) was used in this study. The IHC staining score was calculated by multiplying the staining intensity by the percentage of positive cells. The intensity of IHC staining was determined as: 0 (no staining), 1 (weak staining), 2 (moderate staining), and 3 (strong staining). The percentage of stained cells was determined as: 1 (1–25%), 2 (26–50%), 3 (51–75%), and 4 (76–100%). An IHC score of 4 was determined as the optimal cutoff value analyzed by using SPSS 19.0 (Fig. S9A) and X-tile softwares^58^ (version 3.6.1, Yale University School of Medicine) (Fig. S9B). The IHC staining was scored independently by two pathologists in a blinded manner. Total scores ≥4 were defined as NETO2^high^ and scores <4 were defined as NETO2^low^.

### Quantitative real-time RT-PCR

Quantitative real-time RT-PCR (qRT-PCR) analysis was performed as previously described^59^. Briefly, total RNA was extracted from cell lysates using RNAiso reagent (Takara, Shiga, Japan). Reverse transcription was performed using the PrimeScript^TM^ RT Master Mix Kit (Takara, Dalian, China) following manufacturer’s protocols. qRT-PCR reactions were performed using SYBR Premix Ex Taq II (Takara, Japan) in a Bio-Rad CFX96 Real-Time PCR Detection System (Bio-rad, Hercules, CA, USA) following the manufacturer’s instructions. The relative mRNA levels were normalized against β-actin using the 2^−ΔΔCt^ formula^60^. The primers used in this study were listed in Table S2.

### Bioinformatics analysis

The analyses of NCBI Gene Expression Omnibus (GEO) database (http://www.ncbi.nlm.nih.gov/geo/) were performed as previously described^59^. In brief, the mRNA expression data of NETO2 were downloaded from six GC datasets of GEO database (Accession Numbers: GSE29272, GSE65801, GSE63089, GSE54129, GSE27342, and GSE13911). Downloaded data were log2 transformed and normalized using R software (3.2.5 version, www.r-project.org, R Foundation for Statistical Computing, Vienna, Austria)^61^, and then the expression levels of NETO2 in tumor and normal tissues were compared. The NETO2 expression data in The Cancer Genome Atlas (TCGA) (http://cancergenome.nih.gov/) database were analyzed by using an online tool GEPIA (http://gepia.cancer-pku.cn)^62^. KMPLOT database (http://www.kmplot.com/gastric) was used to evaluate the relationship between NETO2 expression and overall or disease-free survival rates in patients with GC^63^. The prognostic value of NETO2 expression was also analyzed in OncoLnc (www.oncolnc.org), in which both mRNA expression and survival data of 378 GC patients were included.

### Western blotting

Western blotting analyses were performed as previously described^57^. The primary antibodies used in this study were listed in Table S3.

### NETO2 knockdown and overexpression in GC cells

The sequences of two short hairpin RNAs (shRNAs) targeting NETO2 and a scramble (Mock) were listed in Table S4. Lentivirus particles containing sh-NETO2 and mock shRNA were constructed by GeneChem (Shanghai, China) and used to infect XN0422 and MGC803 cells. Stably transfected cells were selected using 3 μg/mL puromycin (Sigma-Aldrich, St. Louis, MO, USA). For overexpressing NETO2 in GC cells, lentiviral particles containing human full-length NETO2 and empty vector were prepared and used to infect XN0422 and MGC803 cells. Stable NETO2-overexpressing (Over-NETO2) cells and control cells were selected using 3 μg/mL puromycin (Sigma, USA).

### Wound healing assay

The wound healing assay was performed as previously described^59^. Briefly, GC cells were pretreated with mitomycin C (1 μg/mL) for 2 h to inhibit cell proliferation. After wounding the cells with pipette tips, the cells were washed twice with PBS and incubated in serum-free RPMI 1640 medium. The wounds were visualized at 0 and 24 h and the migration rate was calculated as the proportion of the closed wound distance compared with the original wound area.

### Transwell invasion assay

Transwell invasion assays were performed as previously described^59^. Briefly, 8.0 µm pore transwell inserts (Millipore, Billerica, MA, USA) were precoated with matrigel and put into 24-well plates. Differently treated GC cells were seeded into the upper chambers (2 × 10^4^/well) and incubated for 24 h. The invaded cells in 5 random fields were counted for each chamber and photographed at 200-fold magnification under a light microscope.

### Intraperitoneal metastasis assay

Intraperitoneal metastasis assay was performed as previously described^57^. Female BALB/c nude mice (5 weeks old) were purchased from the Laboratory Animal Center of the Third Military Medical University (Chongqing, China) and housed in a pathogen-free environment. All animal procedures were approved by the Third Military Medical University Animal Committee and were performed in accordance with the approved University guidelines and regulations. GC cells with different treatments were intraperitoneally injected into nude mice (1 × 10^5^ cells/mouse, 5 mice/group). After 4 weeks, the mice were killed and the metastatic nodules were counted.

### Gene expression profiling and pathway analyses

NETO2-knockdown (sh-NETO2–1) and mock MGC803 cells were used for gene expression profiling. Microarray processing was performed by GeneChem (Shanghai, China) using GeneChip PrimeView Human Gene Expression Array (Affymetrix). *P* values were corrected for multiple testing using Benjamini–Hochberg correction (FDR-corrected *t* tests). Differentially expressed genes between sh-NETO2–1 and mock cells (*n* = 3) were selected by FDR <0.05 and absolute fold change >1.5. The pathway enrichment analysis was performed by using Ingenuity Pathway Analysis (IPA, www.ingenuity.com) software. The *z* score >2 and *z* score <−2 were used as the cutoffs for significant activation and inhibition of pathways, respectively. All primary data are available in Supplementary File 1.

### siRNA and plasmids transfection

The sequences of siRNAs targeting TNFRSF12A and a scramble were designed and synthesized by GeneChem (Shanghai, China) (Table S5). Myristoylated AKT (Myr-AKT) plasmid was purchased from Addgene (Cambridge, MA, USA). Cell transfection was performed using Lipofectamine^TM^ 3000 reagent (Invitrogen, Carlsbad, CA, USA) according to the manufacturer’s instructions.

### Luciferase reporter assay

Luciferase reporter assays were performed as previously described^56^. GC cells were co-transfected with NF-κB luciferase reporter plasmid (pNFκB-Luc, Beyotime, China) and *Renilla* luciferase plasmid (pRL-SV40-C, Beyotime, China) using Lipofectamine^TM^ 3000 transfection reagent (Invitrogen, Carlsbad, CA, USA) according to the manufacturer’s instructions. Reporter activity was analyzed using the Promega dual luciferase assay kit (Promega) following the manufacturer’s instructions. Relative luciferase activity was calculated by normalizing firefly luciferase activity to *Renilla* luciferase activity.

### Statistical analysis

Each experiment was performed at least three times. Statistical analyses were performed using SPSS 19.0 software (IBM SPSS Inc., Chicago, USA) and Prism 5.0 software (GraphPad Software, La Jolla, CA). Differences between two groups were analyzed using Student’s *t*-test. One-way ANOVA was used to compare data containing more than two groups. Besides, data were statistically evaluated using the Pearson *χ*^2^ test, the log-rank test and the multivariate Cox proportional hazards regression analyses. All the values were presented as the mean ± SD. *p* < 0.05 was considered statistically significant.

## Supplementary information

Supplementary tables and figures

Supplementary info
