# Supplementary material for: NETO2 promotes invasion and metastasis of gastric cancer cells via activation of PI3K/Akt/NF-κB/Snail axis and predicts outcome of the patients
Source: Cell Death Dis. 2019 Feb 15;10(3):162. doi: 10.1038/s41419-019-1388-5 (PMC6377647; doi:10.1038/s41419-019-1388-5)
Supplement: Supplementary file 1 — Supplementary tables and figures [file 41419_2019_1388_MOESM1_ESM.docx]

**NETO2 promotes invasion and metastasis of gastric cancer cells via activation of PI3K/Akt/NF-κB/Snail axis and predicts outcome of the patients**

**Authors:** Jun-yan Liu^1^, Lei Jiang^1^, Tao He^1^, Jia-jia Liu^1^, Jun-yan Fan^1^, Xian-hui Xu^1^, Bo Tang^1^, Yan Shi^1^, Yong-liang Zhao^1^, Feng Qian^1^, Yan Wang^2^, You-hong Cui^2, *^, Pei-wu Yu^1,*^

^1^ Department of General Surgery and Center of Minimal Invasive Gastrointestinal Surgery, Southwest Hospital, Third Military Medical University (Army Medical University), Chongqing, 400038, China.

^2^ Institute of Pathology and Southwest Cancer Center, and Key Laboratory of Tumor Immunopathology of Ministry of Education of China, Southwest Hospital, Third Military Medical University (Army Medical University), 400038 Chongqing, China.

^*^Correspondence to: Pei-wu Yu and You-hong Cui. Postal address: Southwest Hospital, Third Military Medical University, No. 30 Gaotanyan Street, Chongqing 400038, China (Pei-wu Yu and You-hong Cui); Tel.: +86 23 68765775 (Pei-wu Yu and You-hong Cui); Fax: +86 23 68765775 (Pei-wu Yu and You-hong Cui); E-mail addresses: [yupeiwu0l@sina.com](mailto:yupeiwu0l@sina.com) (Pei-wu Yu) and [cuiyouhongx@yahoo.com](mailto:cuiyouhongx@yahoo.com) (You-hong Cui).

**Supplementary Tables.**

| **Table S1:** Clinical features of patients with gastric cancer | | |
| --- | --- | --- |
| Clinical Characteristic | Number | Percentage (%) |
| Age |  |  |
| ≥60 | 79 | 35.91 |
| <60 | 141 | 64.09 |
| Sex |  |  |
| Male | 151 | 68.64 |
| Female | 69 | 31.36 |
| Histology grade |  |  |
| G1  G2 | 7 | 3.18 |
| G2 | 63 | 28.64 |
| G3 | 150 | 68.18 |
| T stage |  |  |
| T1 | 16 | 7.27 |
| T2 | 42 | 19.09 |
| T3 | 65 | 29.55 |
| T4 | 97 | 44.09 |
| N stage |  |  |
| N0 | 72 | 32.73 |
| N1 | 47 | 21.36 |
| N2 | 55 | 25.00 |
| N3 | 46 | 20.91 |
| M stage |  |  |
| M0 | 203 | 92.27 |
| M1 | 17 | 7.73 |
| TNM stage |  |  |
| Ⅰ | 33 | 15.00 |
| Ⅱ | 78 | 35.45 |
| Ⅲ | 92 | 41.82 |
| Ⅳ | 17 | 7.73 |
| Tumor size (cm) |  |  |
| ≥5 | 74 | 33.64 |
| <5 | 146 | 66.36 |
| Tumor site |  |  |
| Proximal gastric | 47 | 21.36 |
| Middle gastric | 55 | 25.00 |
| Distal gastric | 118 | 53.64 |

| **Table S2.** Sequences of primers used for qRT-PCR in this study | | |
| --- | --- | --- |
| Gene | Sequence | |
| NETO2 | Forward | 5′- CCACCAAACAAGGAGTGTATCT-3′ |
|  | Reverse | 5′- GGCTTTTCACGCCACAGTAAC-3′ |
| E-cadherin | Forward | 5′-CTACAATGAGCTGCGTGTGG-3′ |
|  | Reverse | 5′-AGGTCCAGACGCAGGATGGC-3′ |
| N-cadherin | Forward | 5′-TCATTGCCATCCTGCTCTGCAT-3′ |
|  | Reverse | 5′-AGTTGTTTGGCCTGGCGTTCTT-3′ |
| Snail | Forward | 5′-GCGCTCTTTCCTCGTCAGG-3′ |
|  | Reverse | 5′-GGGCTGCTGGAAGGTAAACTCT-3′ |
| Twist1 | Forward | 5′-GGCATCACTATGGACTTTCTCTATT-3′ |
|  | Reverse | 5′-GGCCAGTTTGATCCCAGTATT-3′ |
| ZEB1 | Forward | 5′-CAACTACGGTCAGCCCT-3′ |
|  | Reverse | 5′-GCGGTGTAGAATCAGAGTC-3′ |
| TNFRSF12A | Forward | 5′-CTGGCTCCAGAACAGAAAGG-3′ |
|  | Reverse | 5′-GGGCCTAGTGTCAAGTCTGC-3′ |
| β-actin | Forward | 5′-TTGCGTTACACCCTTTCTTG-3′ |
|  | Reverse | 5′-CACCTTCACCGTTCCAGTTT-3′ |

| **Table S3:** Primary antibodies used in this study | | |  |
| --- | --- | --- | --- |
| Target | Dilution | Company | Catalog Number |
| NETO2 | 1:400 | Abcam | ab171651 |
| phospho-Erk1/2 | 1:1000 | CST | 8544 |
| Total-AKT | 1:1000 | CST | 4685 |
| phospho-AKT (Ser473) | 1:2000 | CST | 4060 |
| phospho-AKT (Thr308) | 1:1000 | CST | 4056 |
| phospho-p85 (Tyr458/Tyr199) | 1:1000 | CST | 4228 |
| p85 | 1:1000 | CST | 4257 |
| p110α | 1:1000 | CST | 4255 |
| phospho-NF-κB p65 (Ser536) | 1:1000 | CST | 3033 |
| NF-κB p65 | 1:1000 | CST | 8242 |
| phospho-IKKβ | 1:800 | Abcam | ab59195 |
| IKKβ | 1:1000 | CST | 8943 |
| IκBα | 1:1000 | CST | 9242 |
| phospho-IκBα | 1:1000 | CST | 2859 |
| E-cadherin | 1:1000 | CST | 3195 |
| N-cadherin | 1:1000 | CST | 14215 |
| Snail | 1:1000 | CST | 3879 |
| Twist1 | 1:1000 | CST | 46702 |
| ZEB1 | 1:1000 | CST | 3396 |
| Histon H3 | 1:1000 | CST | 14269 |
| TNFRSF12A | 1:1000 | Abcam | ab109365 |
| β-actin | 1:5000 | Abcam | ab8226 |

| **Table S4.** Sequences of NETO2 knockdown and mock shRNAs used in this study | | |
| --- | --- | --- |
| Gene | Sequence | |
| sh-NETO2-1 | Forward | 5′-CCGGCGCCAAATTATCCTGACTCATCTCGAG ATGAGTCAGGATAATTTGGCG-3′ |
|  | Reverse | 5′-AATTCAAAAACGCCAAATTATCCTGACTCATCTCGAG ATGAGTCAGGATAATTTGGCG-3′ |
| sh-NETO2-2 | Forward | 5′-CCGGCCTCCTCATTATGAACTGTTTCTCGAGAAACAGTTCATAATGAGGAGGTTTTTG-3′ |
|  | Reverse | 5′-AATTCAAAAACCTCCTCATTATGAACTGTTTCTCGAGAAACAGTTCATAATGAGGAGG-3′ |
| mock | Forward | 5′-CCGGTTACGCGTAGCGTAATACGCTCGAG CGTATTACGCTACGCGTAATTTTTG-3′ |
|  | Reverse | 5′-AATTCAAAAATTACGCGTAGCGTAATACGCTCGAG CGTATTACGCTACGCGTAA-3′ |

| **Table S5.** The oligonucleotides used in this study | |
| --- | --- |
| Name | Target Sequence |
| siTNFRSF12A#1 | 5’-CAUCCAUUCUAGAGCCAGUCU-3’ |
| siTNFRSF12A#2 | 5’-GAGGGAGAAUUUAUUAAUAAA-3’ |
| NC | 5’-GCUCAACCGUGAAGUUAUAUU-3’ |

**Supplementary Figures**


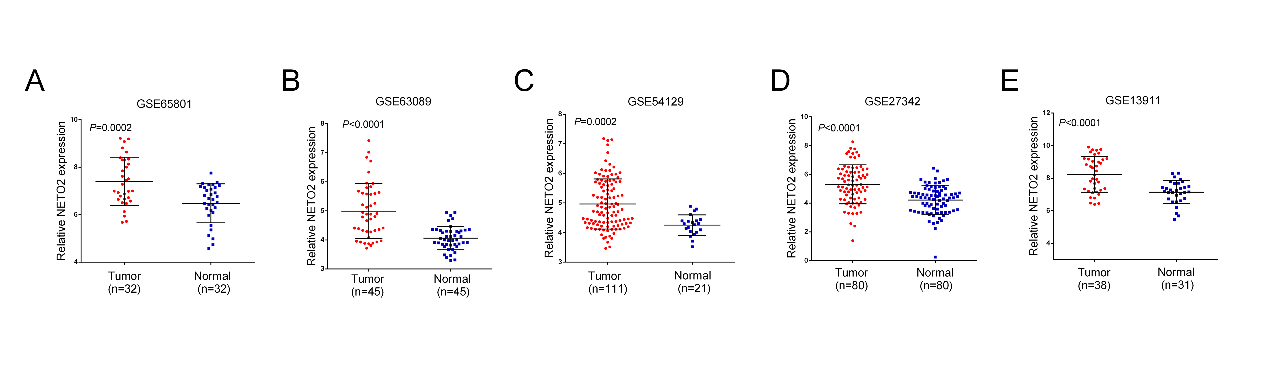


**Figure S1.** Analyses of GEO database showed higher NETO2 expression in gastric cancer tissues than that in normal tissues. (A) Result from GES65801. (B) Result from GSE63089. (C) Result from GSE54129. (D) Result from GSE27342. (E) Result from GSE13911.


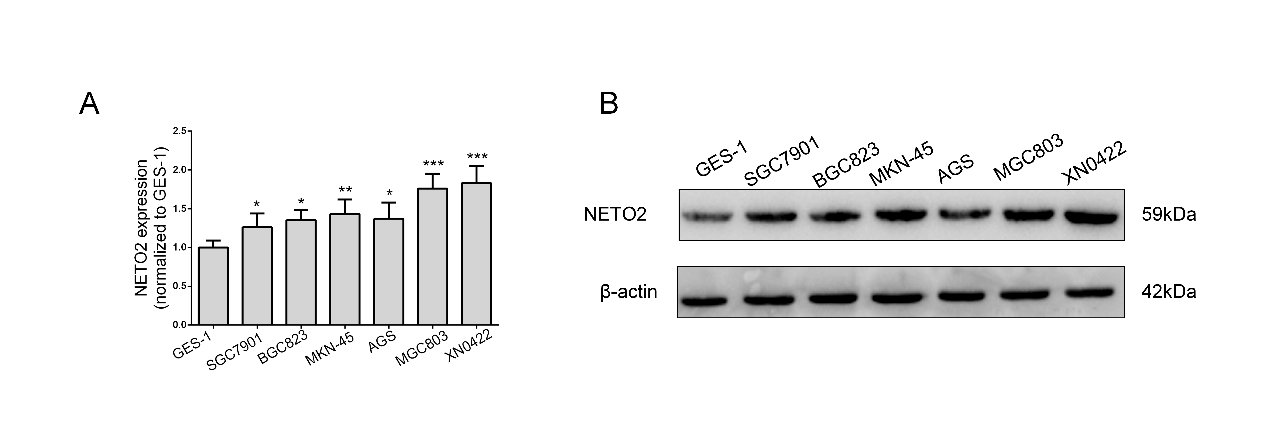


**Figure S2.** Expression levels of NETO2 in 5 gastric cancer cell lines, a primary gastric cell line and a gastric epithelium cell line. (A) mRNA levels of NETO2 in GES-1 (immortalized gastric epithelium cell line), 5 gastric cancer cell lines (SGC7901, BGC823, MKN-45, AGS, MGC803) and a primary gastric cancer cell line (XN0422) analyzed by qRT-PCR. (B) Protein levels of NETO2 in GES-1 (immortalized gastric epithelium cell line), 5 gastric cancer cell lines (SGC7901, BGC823, MKN-45, AGS, MGC803) and a primary gastric cancer cell line (XN0422) analyzed by western blotting.


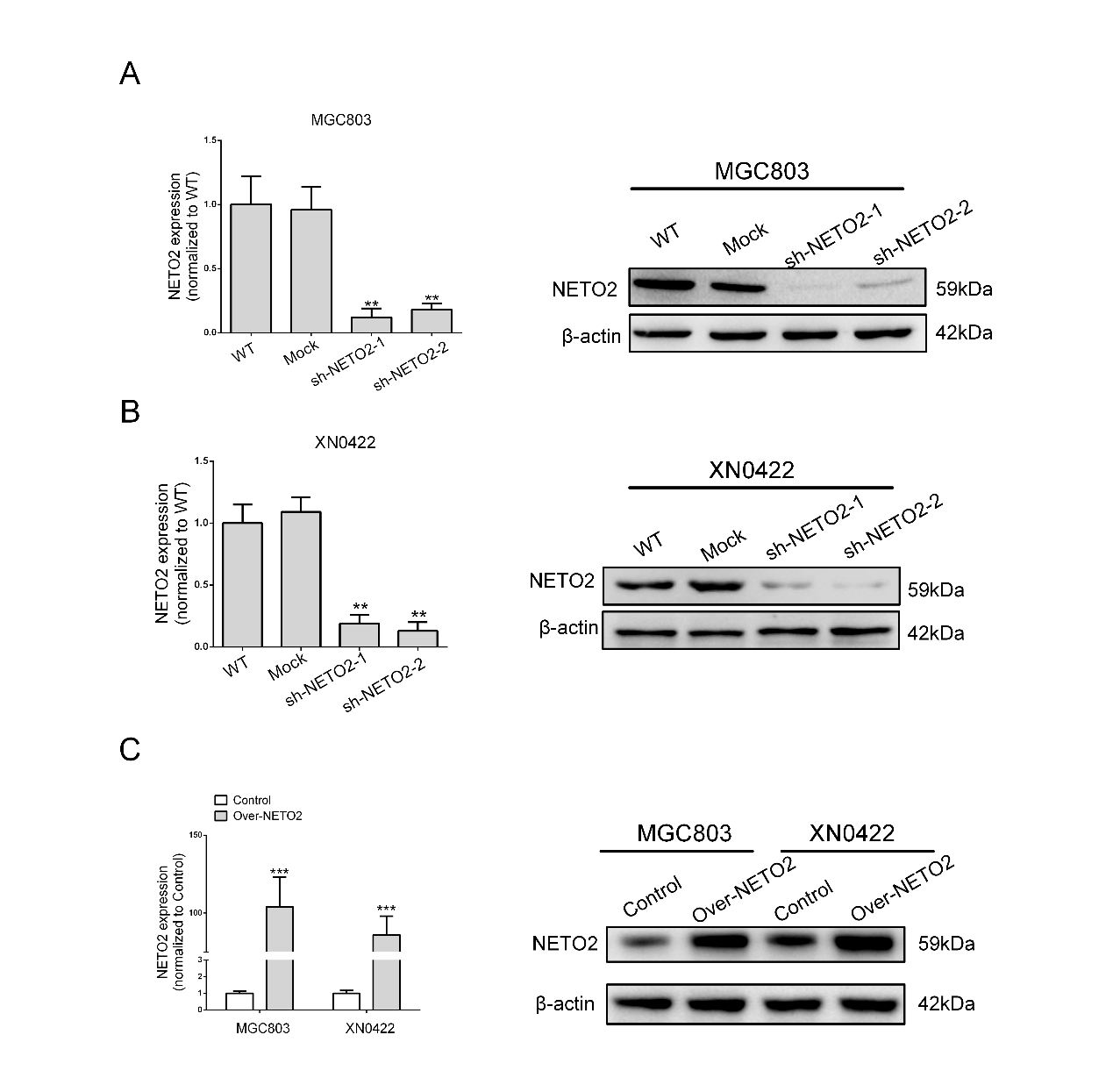


**Figure S3.** The efficiencies of NETO2 knockdown and overexpression in gastric cancer cells. (A) The efficiencies of NETO2 knockdown in MGC803 examined by qRT-PCR and western blotting analyses. (B) The efficiencies of NETO2 knockdown in XN0422 examined by qRT-PCR and western blotting analyses. (C) The efficiencies of NETO2 overexpression in MGC803 and XN0422 examined by qRT-PCR and western blotting.

**
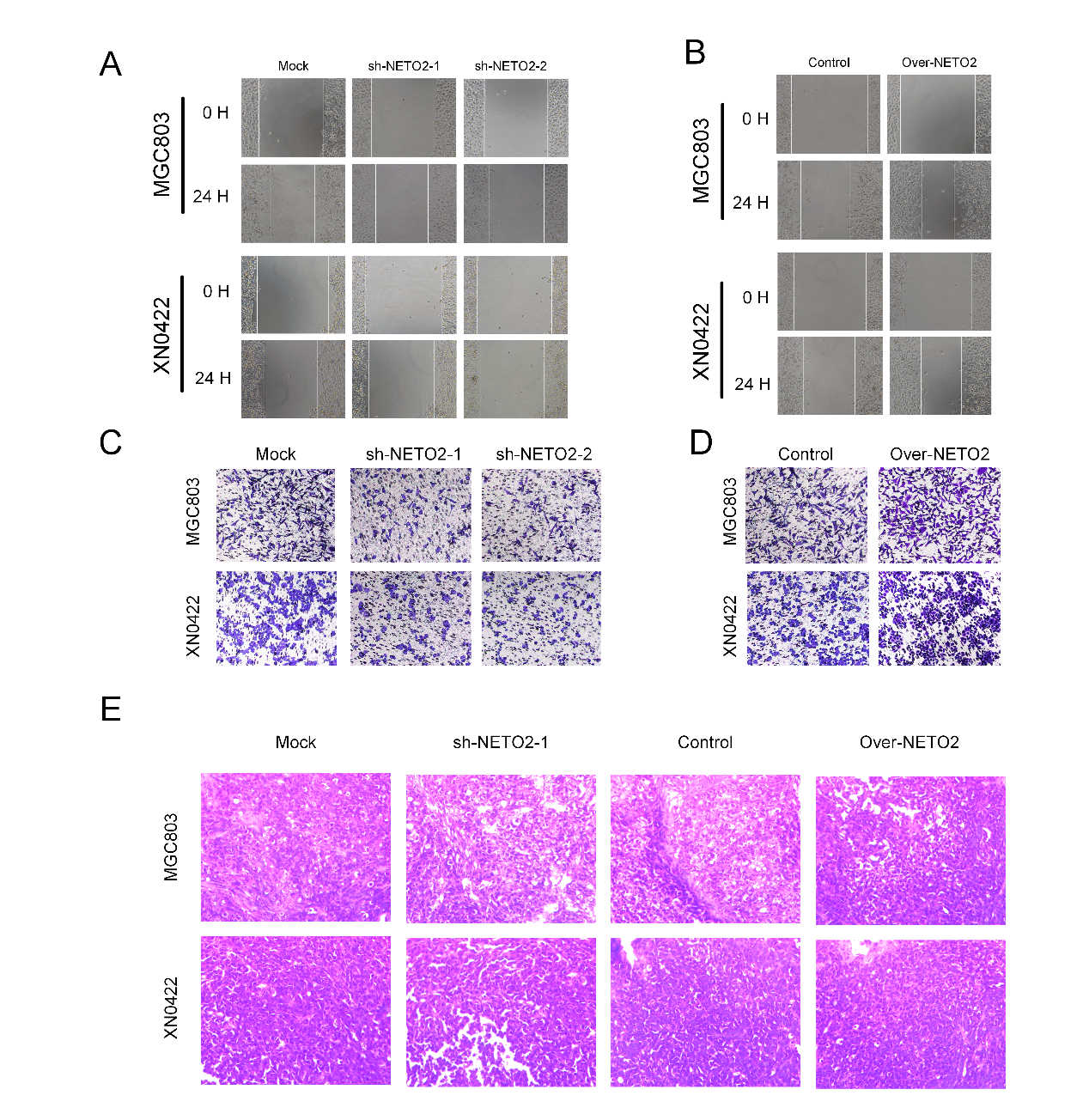
**

**Figure S4.** Representative images of the wound healing and transwell invasion assays for gastric cancer cells with or without NETO2-knockdown and overexpression. (A) Representative images of wound healing assay for NETO2-knockdown MGC803 and XN0422 cells and their mock cells. (B) Representative images of wound healing assay for Over-NETO2 MGC803 and XN0422 cells and their control cells. (C) Representative images of transwell invasion assay for NETO2-knockdown MGC803 and XN0422 cells and their mock cells. (D) Representative images of transwell invasion assay for Over-NETO2 MGC803 and XN0422 cells and their control cells. (E) H&E staining showed the gastric cancer origin of metastatic nodules.


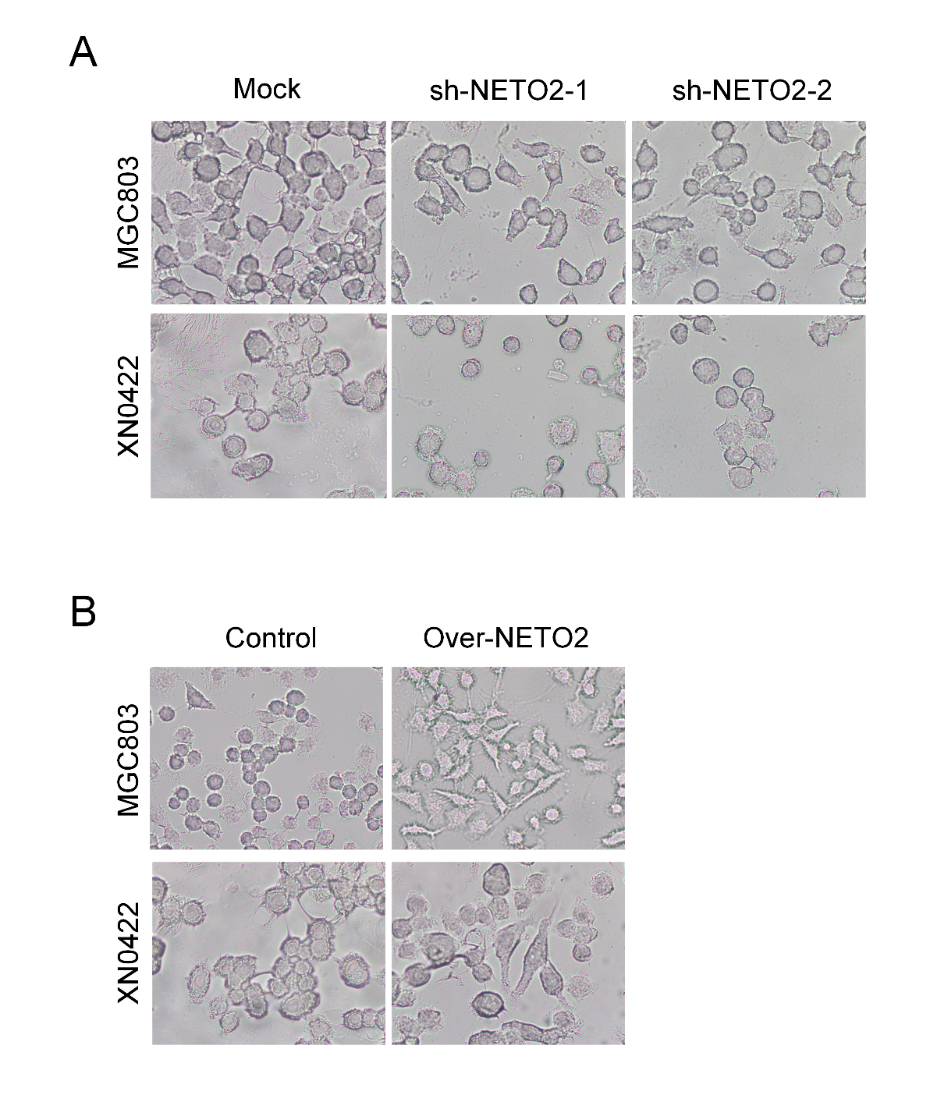


**Figure S5.** Representative images of cell morphology following NETO2 knockdown and overexpression. (A) Cell morphology of mock, sh-NETO2-1 and sh-NETO2-2 gastric cancer cells. (B) Cell morphology of control and Over-NETO2 gastric cancer cells.


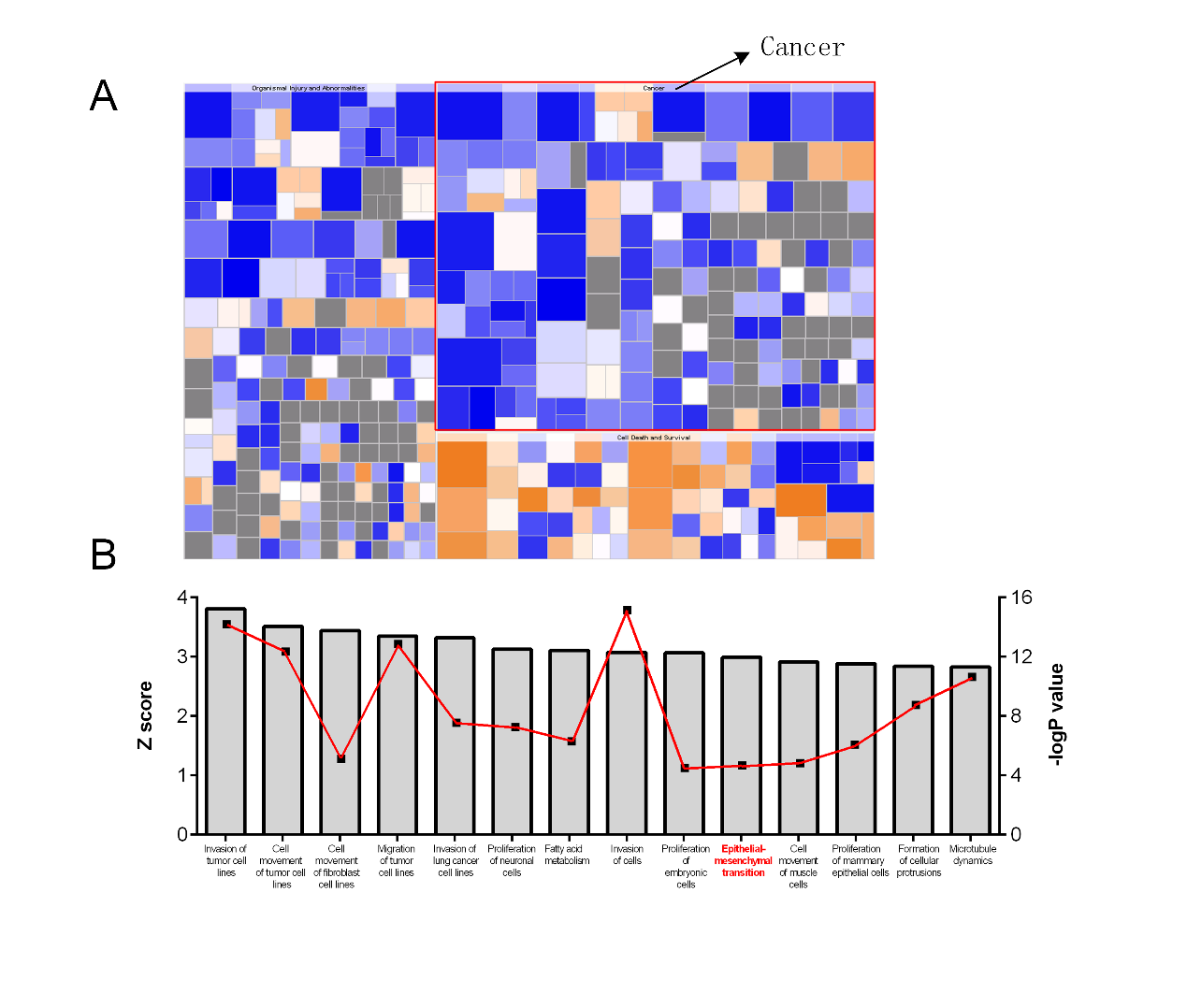


**Figure S6.** The Disease and Function analysis of IPA. (A) Disease and Function heatmap illustrated the relationship between differentially upregulated/downregulated genes and the activated/inhibited functions and diseases. The orange color indicated z score > 0, blue color indicated z score < 0, grey color indicated z score value uncalculated. A z score > 2 indicated that function was strongly activated, whereas a z score < -2 indicated that that function was strongly inhibited. (B) The disease and function histogram illustrated the statistically significant cluster status of the differential genes in categories of disease and function following NETO2 knockdown. All the diseases and functions were ranked by using z score.


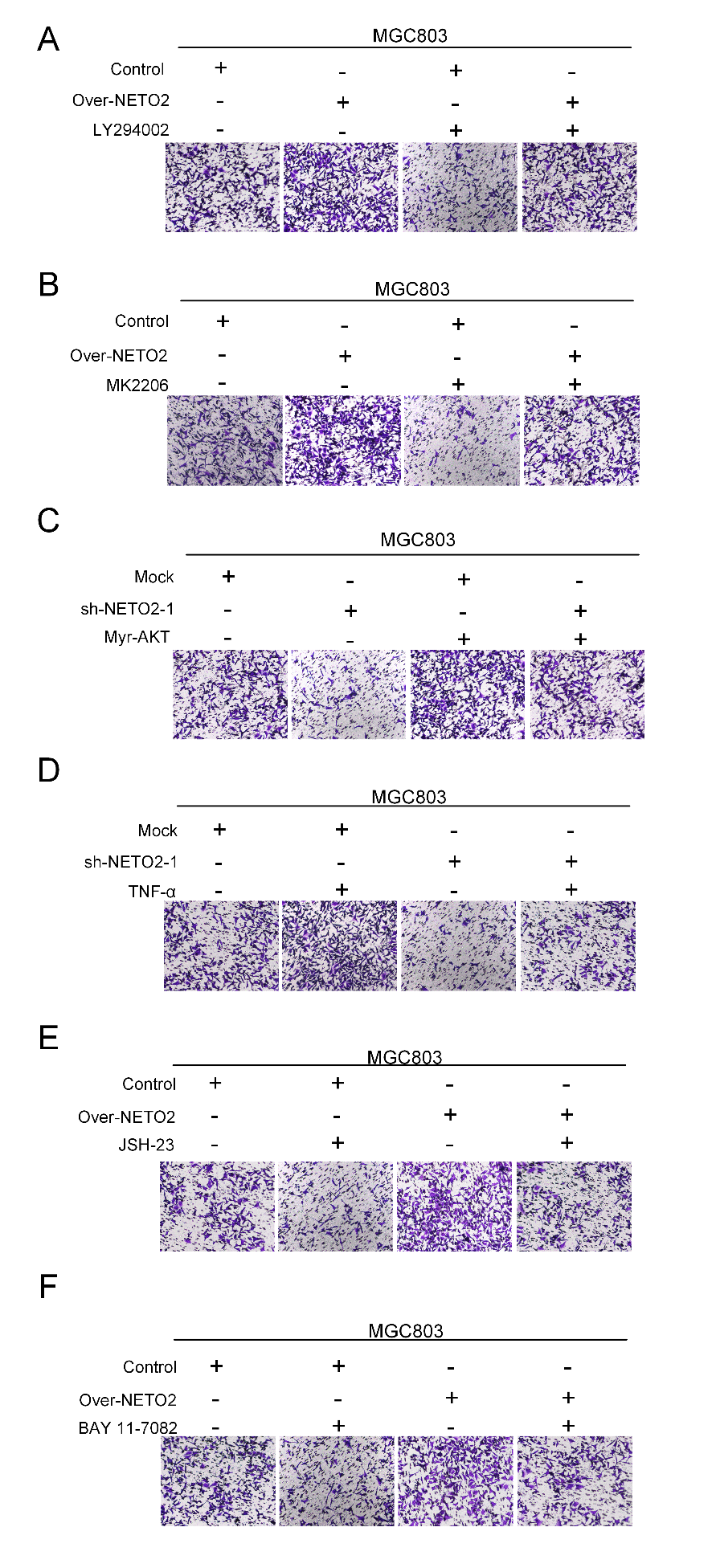


**Figure. S7.** Representative images of transwell invasion assay for gastric cancer cells treated with inhibitors or activators of PI3K, AKT, and NF-kB and transfection with myr-AKT. (A) Representative images of transwell invasion assay for control and Over-NETO2 MGC803 cells treated with or without LY294002 (10μM). (B) Representative images of transwell invasion assay for control and Over-NETO2 MGC803 cells treated with or without MK2206 (5μM). (C) Representative images of transwell invasion assay for mock and sh-NETO2-1 MGC803 cells transfected with or without myr-AKT. (D) Representative images of transwell invasion assay for mock and sh-NETO2-1 MGC803 cells treated with or without TNF-α (10 ng/mL). (E) Representative images of transwell invasion assay for control and Over-NETO2 MGC803 cells treated with or without JSH-23 (10μM). (F) Representative images of transwell invasion assay for control and Over-NETO2 MGC803 cells treated with or without BAY 11-7082 (10μM).

**
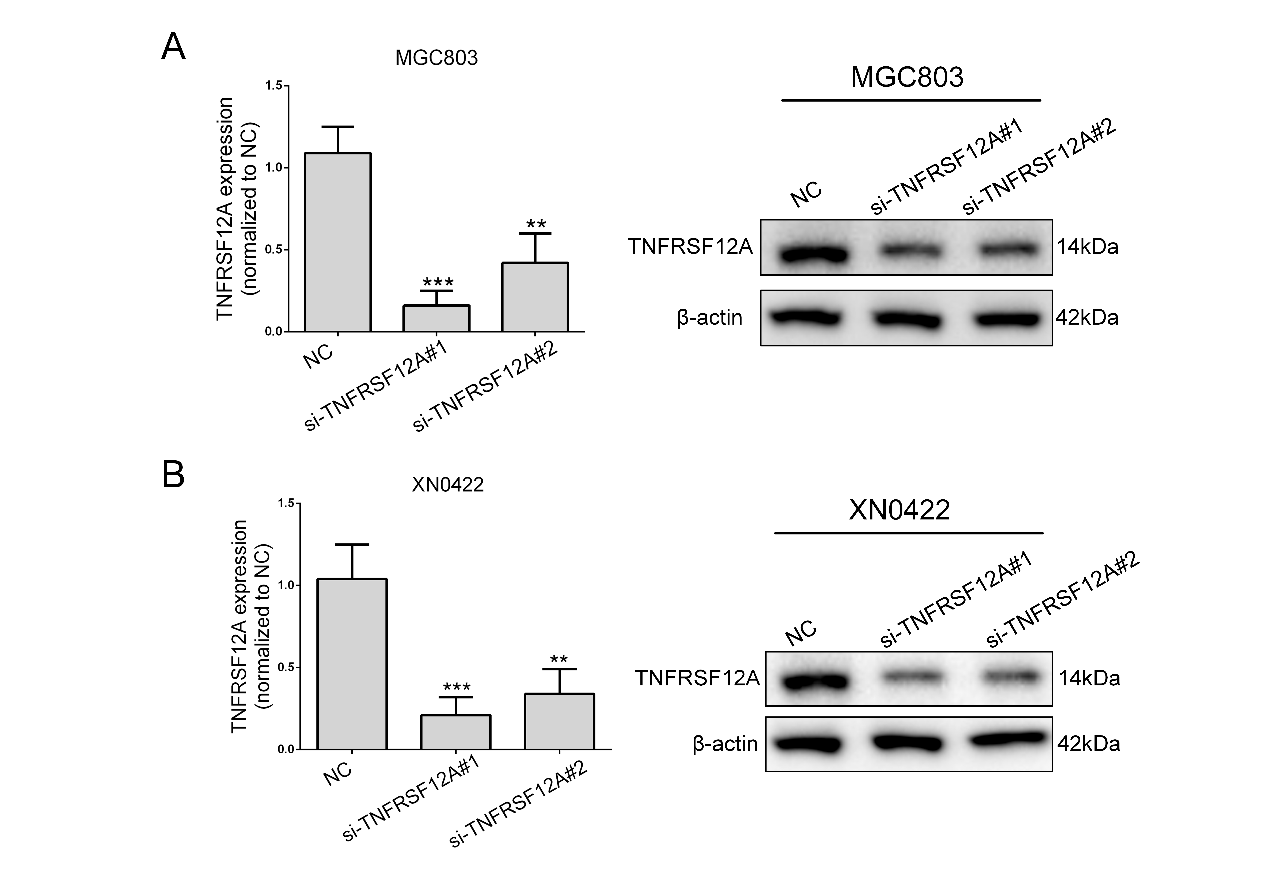
**

**Figure S8.** The efficiencies of TNFRSF12A knockdown by siRNA in MGC803 and XN0422 cells. (A) The knockdown efficiencies of TNFRSF12A in MGC803 cells analyzed by qRT-PCR and western blotting. (B) The knockdown efficiencies of TNFRSF12A in XN0422 cells analyzed by qRT-PCR and western blotting.

**
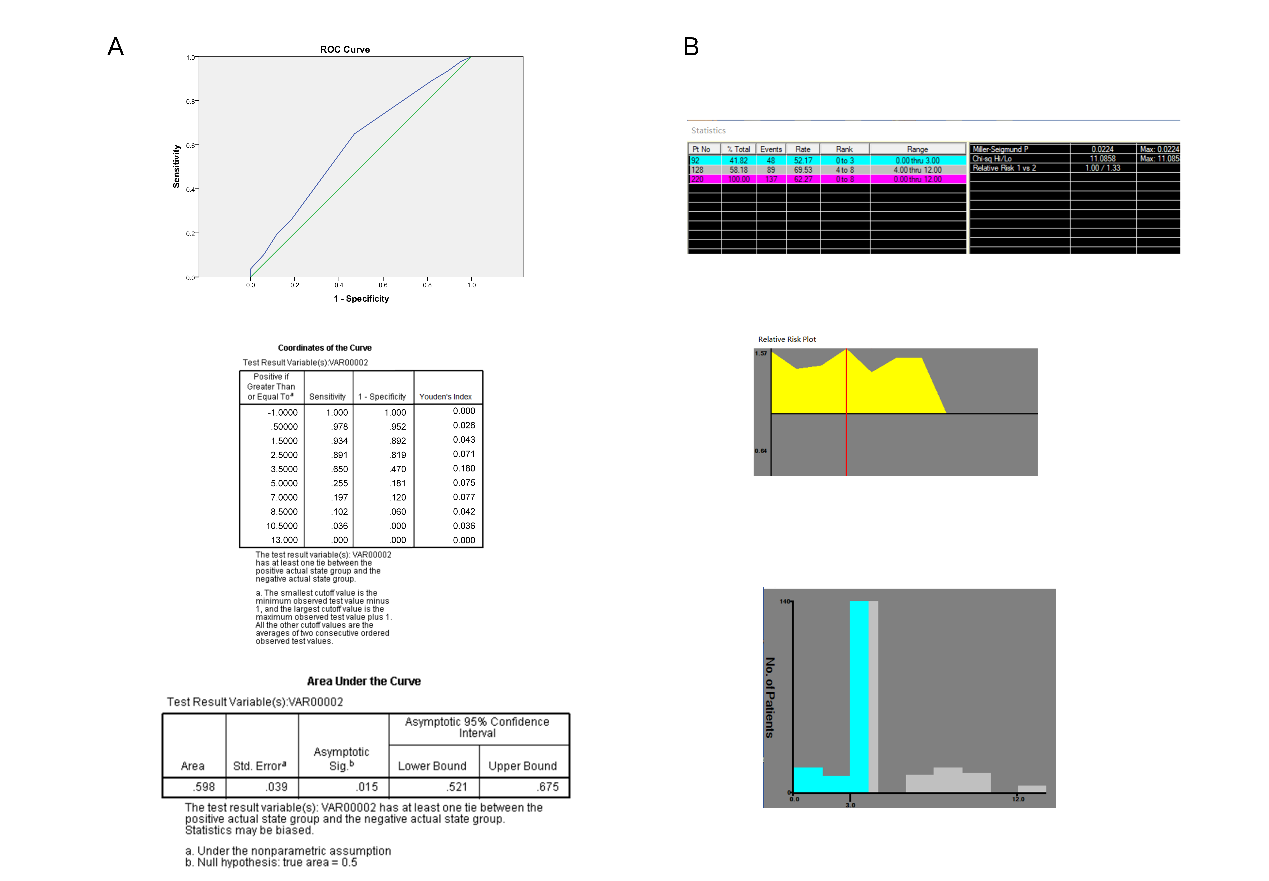
**

**Figure S9.** The determination of the optimal cutoff value for the IHC staining of NETO2 in 220 patients with gastric cancer. (A) The ROC curve and Youden’s index analyzed by SPSS 19.0 software. The area under the curve was 0.598 (*p* = 0.015). The maximum value of Youden’s index was 0.180 when the immunohistochemical score was 3.5. (B) Analysis with X-tile software showed the optimal cutoff value was 4.
